# Posterior atlantoaxial fixation with new subfacetal axis screw trajectory avoiding vertebral artery with customized variable screw placement plate and screws to enhance biomechanics of fixation

**DOI:** 10.3171/2020.4.FocusVid.20168

**Published:** 2020-07-01

**Authors:** Sushil Patkar

**Affiliations:** Department of Neurosurgery, Poona Hospital and Research Center, Pune, Maharashtra, India

**Keywords:** atlantoaxial dislocation, atlantoaxial fixation, vertebral artery injury

## Abstract

Fixation for atlantoaxial dislocation is a challenging issue, and posterior C1 lateral mass and C2 pars–pedicle screw plate–rod construct is the standard of care for atlantoaxial instability. However, vertebral artery injury remains a potential complication. Recent literature has focused on intraoperative navigation, the O-arm, 3D printing, and recently use of robots for perfecting the trajectory and screw position to avoid disastrous injury to the vertebral artery and enhance the rigidity of fixation. These technological advances increase the costs of the surgery and are available only in select centers in the developed world.

Review of the axis bone anatomy and study of the stress lines caused by weight transmission reveal that the bone below the articular surface of the superior facet is consistently dense as it lies along the line of weight transmission A new trajectory for the axis screw 3–5 mm below the midpoint of the facet joint and directed downward and medially avoids the course of the vertebral artery and holds the axis rigidly. Divergent screw constructs are biomechanically stronger. Variable screw placement (VSP) plates with long shaft screws permit manipulation of the vertebrae and realignment of the facets to the correct reduced position with fixation in the compression mode.

The video can be found here: https://youtu.be/E1msiKjM-aA

## Transcript

**0:20** Introduction. Atlantoaxial dislocation is a challenging problem. C1 lateral mass with C2 pars–pedicle plate–rod fixation is the current standard of care. Avoiding vertebral artery injury and correction of the deformity are the two main concerns which need attention. Recent literature has focused on the use of intraoperative navigation, O-arm, and 3D printing to enhance safety, but these come at an increased cost (Goel and Laheri, 1994; Harms and Melcher, 2001).

Review of the axis vertebra anatomy consistently shows the presence of dense compact bone below the C2 facet, which has been confirmed by recent publications in the literature (Menon and Raniga, 2018).

**1:30** The new trajectory. Starting the C2 fixation few millimeters below the midpoint of the superior facet eliminates the chances of vertebral artery injury.

Directing the screw downward and medially provides for a strong hold for manipulation and rigid fixation.

**1:54** Cadaveric dissection and live operative clips. In this cadaveric dissection, after standard posterior midline exposure, the occiput, posterior arch of the atlas, and posterior element of axis along with both atlantoaxial joints are exposed.

The new entry point for the axis screw is few millimeters below the midpoint of the facet joint, and the trajectory is downward and medially in the subfacetal region of the axis for a depth of 16–20 mm. This is decided by studying the preoperative CT scan for bone stock and volume. The tap is used to thread the drill holes for a few millimeters to allow for screw entry and direction. Overtapping must be avoided (Patkar SV, 2016).

The C1 screw is above and lateral to the midpoint of the facet joint and directed upward and medially, divergent with respect to the axis screw. The length again is decided on preoperative CT scans and bone stock. The joint space is gardened by abrading the endplate with a microburr and curette, followed by corticocancellous autografting with bone harvested from the spinous process of the axis and C3.

**3:57** Preop images. These preoperative images show atlantoaxial instability, both in flexion and extension with a high-riding vertebral artery.

**4:09** New screw and VSP plate construct and facetal realignment. The new screws with long shafts and variable screw placement plates permit realignment of the vertebra by using joysticks and by fixing the top nut at the appropriate position on the plate. The VSP plate construct permits alignment of the screws in the VSP plate in the desired position in compression mode, which is difficult in the polyaxial screw-rod construct. The extra length of the screw is cut, and now the rigid construct is seen with the screws fixed in compression mode.

**5:40** The biomechanics of divergent screws. Divergent screw direction is an important concept which results in better fixation. These are the special instruments made for the VSP plate-screw construct.

**6:01** Postoperative images and follow-up images. These postoperative images show correct realignment and screw position. These postoperative images after 6 months show solid bony fusion.

**6:15** Additional references. Li et al., 2015; Patkar, 2016; Menon and Raniga, 2018.
